# Mediating Factors in the Association of Maternal Educational Level With Pregnancy Outcomes: A Mendelian Randomization Study

**DOI:** 10.1001/jamanetworkopen.2023.51166

**Published:** 2024-01-11

**Authors:** Tormod Rogne, Dipender Gill, Zeyan Liew, Xiaoting Shi, Vilde Hatlevoll Stensrud, Tom Ivar Lund Nilsen, Stephen Burgess

**Affiliations:** 1 Department of Chronic Disease Epidemiology, Yale School of Public Health, New Haven, Connecticut; 2 Center for Perinatal, Pediatric and Environmental Epidemiology, Yale School of Public Health, New Haven, Connecticut; 3 Department of Epidemiology and Biostatistics, School of Public Health, Imperial College London, London, United Kingdom; 4 Department of Environmental Health Sciences, Yale School of Public Health, New Haven, Connecticut; 5 Department of Public Health and Nursing, Norwegian University of Science and Technology, Trondheim, Norway; 6 Clinic of Anesthesia and Intensive Care, St Olav’s Hospital, Trondheim University Hospital, Trondheim, Norway; 7 Medical Research Council Biostatistics Unit, University of Cambridge, Cambridge, United Kingdom; 8 Cardiovascular Epidemiology Unit, Department of Public Health and Primary Care, University of Cambridge, Cambridge, United Kingdom

## Abstract

**Importance:**

Lower educational attainment is associated with increased risk of adverse pregnancy outcomes, but it is unclear which pathways mediate this association.

**Objective:**

To investigate the association between educational attainment and pregnancy outcomes and the proportion of this association that is mediated through modifiable cardiometabolic risk factors.

**Design, Setting, and Participants:**

In this 2-sample mendelian randomization (MR) cohort study, uncorrelated (*R^2^* < 0.01) single-nucleotide variants (formerly single-nucleotide polymorphisms) associated with the exposure (*P* < 5 × 10^−8^) and mediators and genetic associations with the pregnancy outcomes from genome-wide association studies were extracted. All participants were of European ancestry and were largely from Finland, Iceland, the United Kingdom, or the US. The inverse variance–weighted method was used in the main analysis, and the weighted median, weighted mode, and MR Egger regression were used in sensitivity analyses. In mediation analyses, the direct effect of educational attainment estimated in multivariable MR was compared with the total effect estimated in the main univariable MR analysis. Data were extracted between December 1, 2022, and April 30, 2023.

**Exposure:**

Genetically estimated educational attainment. The mediators considered were genetically estimated type 2 diabetes, body mass index, smoking, high-density lipoprotein cholesterol level, and systolic blood pressure.

**Main Outcomes and Measures:**

Ectopic pregnancy, hyperemesis gravidarum, gestational diabetes, preeclampsia, preterm birth, and offspring birth weight.

**Results:**

The analyses included 3 037 499 individuals with data on educational attainment, and those included in studies on pregnancy outcomes ranged from 141 014 for ectopic pregnancy to 270 002 with data on offspring birth weight. Each SD increase in genetically estimated educational attainment (ie, 3.4 years) was associated with an increased birth weight of 42 (95% CI, 28-56) g and an odds ratio ranging from 0.53 (95% CI, 0.46-0.60) for ectopic pregnancy to 0.81 (95% CI, 0.71-0.93) for preeclampsia. The combined proportion of the association that was mediated by the 5 cardiometabolic risk factors ranged from −17% (95% CI, −46% to 26%) for hyperemesis gravidarum to 78% (95% CI, 10%-208%) for preeclampsia. Sensitivity analyses accounting for pleiotropy were consistent with the main analyses.

**Conclusions and Relevance:**

In this MR cohort study, intervening for type 2 diabetes, body mass index, smoking, high-density lipoprotein cholesterol level, and systolic blood pressure may lead to reductions in several adverse pregnancy outcomes associated with lower levels of education. Such public health interventions would serve to reduce health disparities attributable to social inequalities.

## Key Points

**Question:**

Which pathways mediate the inequity in pregnancy health associated with low educational attainment?

**Findings:**

In this cohort study of more than 3 million individuals, an association between genetically estimated lower educational attainment and increased risk of ectopic pregnancy, hyperemesis gravidarum, gestational diabetes, preeclampsia, preterm birth, and offspring low birth weight was observed. A sizeable portion of these associations were explained by targetable risk factors.

**Meaning:**

These findings suggest that the association of socioeconomic inequalities with adverse pregnancy outcomes may be reduced by intervening for type 2 diabetes, body mass index, smoking, high-density lipoprotein cholesterol level, and systolic blood pressure.

## Teaser

This cohort study evaluates the mediating pathways underlying the association between educational attainment and pregnancy complications and including ectopic pregnancy, hyperemesis gravidarum, gestational diabetes, preeclampsia, preterm birth, and offspring birth weight.

## Introduction

Socioeconomic factors—educational attainment in particular—are associated with adverse pregnancy outcomes.^1,2,3,4,5^ However, it is challenging to modify an individual’s level of education, and opportunities to seek education are not equally distributed throughout the population. It is therefore of great importance to identify modifiable risk factors through which the association with educational attainment is mediated.^6^

Conventional observational studies have identified some potential mediating pathways, and cardiometabolic risk factors stand out.^1^ For instance, prepregnancy body mass index (BMI) and systolic blood pressure (SBP) have been observed to explain most of the association between educational level and gestational hypertension and preeclampsia.^2,3^ Smoking has been observed to likely mediate some of the potential association of educational level with preterm birth^7^ and low birth weight,^4^ but not with the risk of preeclampsia.^2^ For preterm birth, most of the likely association with educational attainment remains unexplained.^1^ For yet other pregnancy outcomes, such as ectopic pregnancy, hyperemesis gravidarum, and gestational diabetes, the role of any mediating pathways downstream of educational attainment remains largely unknown.^5,8,9^ Thus, there is a need for a systematic evaluation of targetable risk factors that may help reduce socioeconomic inequalities in pregnancy outcomes.

There are 2 other important limitations in the literature on educational attainment and pregnancy outcomes. First, many studies lack adjustment for key confounders,^1^ and residual confounding may have biased the results.^10^ Second, mediation analyses in traditional observational studies are susceptible to measurement error, such as day-to-day variations of a mediator, which in turn underestimates the mediating effect.^11^

Mendelian randomization (MR) studies use genetic variants as instruments to evaluate the association between an exposure and an outcome. Because genetic variants are allocated at random and are not influenced by lifestyle factors and chronic conditions, MR studies are robust to bias by both measured and unmeasured confounders. Furthermore, because genetic variants serve as proxies for the long-term effect of an exposure or mediator, MR studies are generally robust to nondifferential measurement error.^12^

We aimed to conduct the first MR study, to our knowledge, to evaluate the mediating pathways underlying the association between educational attainment and pregnancy complications. Specifically, we focused on 6 pregnancy complications and outcomes that are common and/or severe: ectopic pregnancy, hyperemesis gravidarum, gestational diabetes, preeclampsia, preterm birth, and offspring birth weight. For the mediating factors, we followed the statement by the American Heart Association on optimizing pregnancy health^13^ and investigated the mediating role of type 2 diabetes (T2D), BMI, smoking, high-density lipoprotein cholesterol (HDL-C) level, and SBP.

## Methods

### Study Design

In this 2-sample MR cohort study, we used publicly available, summary-level data with relevant ethical approvals, which did not require institutional review board approval or informed consent. The study followed the Strengthening the Reporting of Observational Studies in Epidemiology (STROBE) reporting guideline for MR.^14^ In a 2-sample MR study, summary level data from genome-wide association studies (GWAS) were used to find genetic proxies of an exposure and investigate the associations of these proxies with an outcome (detailed in the eMethods and eFigure in Supplement 1).^10^ Data were extracted between December 1, 2022, and April 30, 2023.

### Instrumental Variable Selection for Educational Attainment

Genetic instruments for educational level were extracted from a GWAS by Okbay et al^15^ (Table), the largest GWAS on educational attainment at the time of analysis. In that GWAS, years of education were standardized in the participating cohorts by mapping the highest level of education to an International Standard Classification of Education 1997 category. The mean (SD) level of education was 15.4 (3.4) years. We extracted single-nucleotide variants (SNVs; formerly SNPs) that were strongly associated with educational attainment, defined as *P* < 5 ×10^−8^, and that were independent of one another, defined as a pairwise *R*^2^ < 0.01 based on the 1000 Genomes Project European ancestry superpopulation.

**Table.** GWAS Used as Sources for 2-Sample Mendelian Randomization Analyses

Trait	Source	Setting	Country	Phenotype definition	No. of participants or No. of cases/controls
Exposure					
Educational level	Okbay et al,^15^ 2022	71 Cohorts, including UK Biobank and 23andMe	Many, including UK and US	Self-reported years of education	3 037 499
Mediators					
T2D	Mahajan et al,^16^ 2018	32 Cohorts, including deCODE and UK Biobank	Many, including Iceland and UK	Cases: T2D status based on a combination of diagnostic testing (fasting glucose or HbA_1c_ level), recorded diagnosis codes, or self-report; controls: not diagnosed with T2D	74 124/824 006
BMI	Pulit et al,^17^ 2019	30 Cohorts, including UK Biobank	Many, including UK	Measured BMI at study participation	806 834
Lifetime smoking score	Wootton et al,^18^ 2020	UK Biobank	UK	Self-reported smoking behavior, lifetime smoking index constructed to reflect smoking status (ever vs never), and smoking intensity among ever smokers	462 690
HDL-C level	Graham et al,^19^ 2021	146 Cohorts, including deCODE, Million Veteran Program, and UK Biobank	Many, including Iceland, UK, and US	Measured blood levels of HDL-C level	1 244 580
SBP	Neale Laboratories, release 2^8^	UK Biobank	UK	Automated reading of SBP	340 159
Outcomes					
Ectopic pregnancy	FinnGen, release 8^20^	FinnGen	Finland	Cases: *ICD-8* code 631, *ICD-9* code 633, or *ICD-10* code O00; controls: women without mentioned *ICD* codes	5052/135 962
Hyperemesis gravidarum	FinnGen, release 8^20^	FinnGen	Finland	Cases: *ICD-8* code 638, *ICD-9* code 643, or *ICD-10* code O21; controls: women without mentioned *ICD* codes	2092/163 702
Gestational diabetes	FinnGen, release 8^20^	FinnGen	Finland	Cases: *ICD-9* code 6488A or *ICD-10* code O24.4; controls: women without mentioned *ICD* codes	11 279/179 600
Preeclampsia	Steinthorsdottir et al,^21^ 2020	6 Cohorts, including deCODE	Many, including Iceland	Cases: maternal, varied by cohort (GOPEC, ALSPAC, and MOBA, pregnancy-onset hypertension and proteinuria; deCODE, *ICD-10* codes O13, O14 or O15; SSI, *ICD-8* code 637.04 and *ICD-10* codes 014.1, O14.2 and O15; and FINRISK, *ICD-8* codes 637.03, 637.04, 637.09, 637.10, 637.99, *ICD-9* codes 6424-6427A, and *ICD-10* codes O14.0, O14.1, O14.9, O15.0, O15.1, O15.2, and O15.9); controls: nonpreeclampsia pregnancies, except GOPEC and deCODE, which used women without preeclampsia	7219/155 660
Preterm birth	Solé-Navais et al,^22^ 2023	18 Cohorts, including deCODE and UK Biobank	Many, including Iceland and UK	Singleton live birth with spontaneous onset; cases: delivery <259 d or *ICD-10* code O60; controls: delivery between 273 to 294 d	15 419/217 871
Birth weight	Juliusdottir et al,^23^ 2021	23 Cohorts, including deCODE and UK Biobank	Many, including Iceland and UK	Maternal GWAS of offspring birth weight	270 002

Abbreviations: ALSPAC, Avon Longitudinal Study of Parents and Children; BMI, body mass index; GOPEC, UK Genetics of Pre-eclampsia study; GWAS, genome-wide association study; HbA_1c_, hemoglobin A_1c_; HDL-C, high-density lipoprotein cholesterol; *ICD-8*, *International Classification of Diseases, Eighth Revision*; *ICD-9*, *International Classification of Diseases, Ninth Revision*; *ICD-10*, *International Statistical Classification of Diseases and Related Health Problems, Tenth Revision*; MOBA, Norwegian Mother and Child Cohort Study; SBP, systolic blood pressure; SSI, Statens Serum Institut; T2D, type 2 diabetes; UK, United Kingdom.

### Outcomes

Genetic associations for 3 of the outcomes—ectopic pregnancy, hyperemesis gravidarum, and gestational diabetes—were extracted from a database of publicly available GWAS summary statistics from FinnGen (Table).^20^ For preeclampsia, we used data from a study by Steinthorsdottir et al^21^; for preterm birth, a study by Solé-Navais et al^22^; and for offspring birth weight, a study by Juliusdottir et al.^23^ All pregnancy outcomes were binary, except for birth weight, which was analyzed as a continuous outcome.

### Mediators

We chose 5 cardiometabolic traits from the list of targetable risk factors to optimize pregnancy health according to the American Heart Association’s 2023 scientific statement.^13^ Single-nucleotide variant effects on the risk of T2D were extracted from a study by Mahajan et al^16^; on BMI, from a study by Pulit et al^17^; and on HDL-C level, from a study by Graham et al.^19^ For smoking behavior, we used a GWAS by Wootton et al^18^ on a lifetime smoking index that reflects a combination of smoking status (ever vs never) and duration, heaviness, and cessation. Finally, we used GWAS results from UK Biobank created by the Neale Laboratory (release number 2)^24^ that evaluated SNVs without adjustments for BMI. Type 2 diabetes was the only binary mediator, while the others were continuous (Table).

### Statistical Analysis

To address the aims of this study, we sought to evaluate the following measures: (1) the association of educational attainment with each pregnancy outcome, (2) the association of educational attainment with each cardiometabolic risk factor, (3) the association of each cardiometabolic risk factor with each pregnancy outcome, and (4) the direct association of educational attainment with each pregnancy outcome after accounting for each cardiometabolic risk factor separately and combined. The first 3 measures were based on univariable MR analyses, while the fourth measure was based on multivariable MR analyses. Under the instrumental variable assumptions, the univariable MR estimate represents the total effect of the exposure, whereas the multivariable MR estimate represents the direct effect of the exposure (ie, the effect of intervening on the exposure but holding all mediators constant).

Prior to analyses, we harmonized the files to ensure that the effect estimate of a given SNV was oriented to the same allele in all files. The threshold for statistical significance was *P* < .05, and the tests were 2 sided. All analyses were run using R, version 4.2.0 (R Program for Statistical Computing) and the R packages MendelianRandomization, version 0.7.0, TwoSampleMR, version 0.5.6, and metaphor, version 3.4.0.

#### Univariable MR Analysis

For each association (eg, educational attainment and birth weight), we calculated the Wald ratio per SNV and used inverse variance–weighted (IVW) analysis to summarize the associations of all SNVs, which puts more emphasis on the estimates with the lowest variance.^10^ For the IVW estimate to be unbiased, however, all SNVs included in the analysis must be valid. There are 3 key assumptions that must be met for an instrument to be valid: It must be associated with the exposure; it cannot be associated with a confounder of the exposure-outcome association; and it is not associated with the outcome other than through the exposure (eFigure in Supplement 1).^10^ For all univariable MR analyses, we conducted 3 sensitivity analyses that provide unbiased estimates even in the presence of some invalid instruments: the weighted median, weighted mode, and MR Egger regression (eTable 1 in Supplement 1).^10^

#### Mediation Analysis

We calculated the direct association with educational attainment on each pregnancy outcome by conducting multivariable MR analyses with each of the 5 cardiometabolic mediators at a time, then with all mediators combined.^10,25^ The total association was provided by the aforementioned univariable MR analyses. To calculate the proportion mediated, we divided the direct by the total association and subtracted from 1. Finally, we estimated the SEs using bootstrapping.^26^ The mediation analyses were based on the estimates from the IVW analyses.

## Results

### Overall Association Among Genetically Estimated Educational Attainment, Cardiometabolic Mediators, and Pregnancy Outcomes

**Figure 1.**
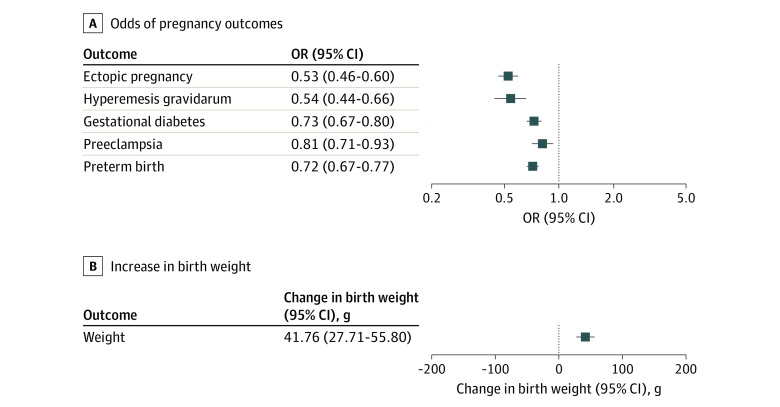
Associations Between Genetically Estimated Educational Attainment and Pregnancy Outcomes Results of inverse variance–weighted 2-sample mendelian randomization analyses are shown. Estimates are odds ratios (ORs) for pregnancy outcomes and grams of birth weight per 1-SD increase of genetically estimated years of education (3.4 years).

The GWAS on educational attainment evaluated 3 037 499 individuals (from 71 cohorts), and the number of individuals included in the studies on pregnancy outcomes ranged from 141 014 (ectopic pregnancy, from 1 cohort) to 270 002 (birth weight, from 23 cohorts). The genetic instruments for educational attainment explained 7.5% of its variance, with median *F* statistic for the individual SNVs of 49 (range, 28-576). There was a protective association of a higher level of genetically estimated educational attainment with all pregnancy outcomes (range of odds ratios [ORs], 0.53 [95% CI, 0.46-0.60] for ectopic pregnancy to 0.81 [95% CI, 0.71-0.93] for preeclampsia) and a positive association with increased offspring birth weight (42 [95% CI, 28-56] g) (Figure 1). These associations were robust in sensitivity analyses that evaluated potential bias due to genetic pleiotropy (eTable 2 in Supplement 1).

**Figure 2.**
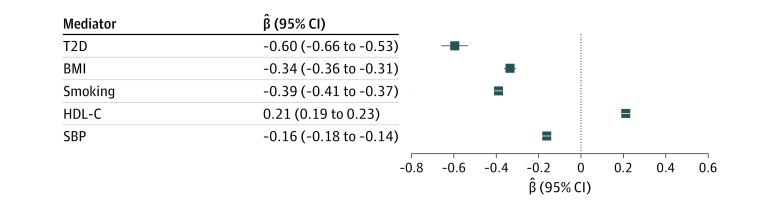
Associations Between Genetically Estimated Educational Attainment and Cardiometabolic Mediators Results of inverse variance–weighted 2-sample mendelian randomization analyses are shown. Estimates are the change in the mediator per 1-SD increase of genetically estimated years of education (3.4 years), and the mediators are in log(odds) units for type 2 diabetes (T2D) and in SD units for the other cardiometabolic risk factors. BMI indicates body mass index; HDL-C, high-density lipoprotein cholesterol level; and SBP, systolic blood pressure.

Educational attainment was also associated with each of the considered cardiometabolic mediators (β coefficient range, −0.06 [95% CI, −0.66 to −0.53] for T2D to 0.21 [95% CI, 0.19-0.23] for HDL-C level) (Figure 2). This finding was supported by the sensitivity analyses accounting for pleiotropy (eTable 3 in Supplement 1).

### Association Between Genetically Estimated Cardiometabolic Mediators and Pregnancy Outcomes

**Figure 3.**
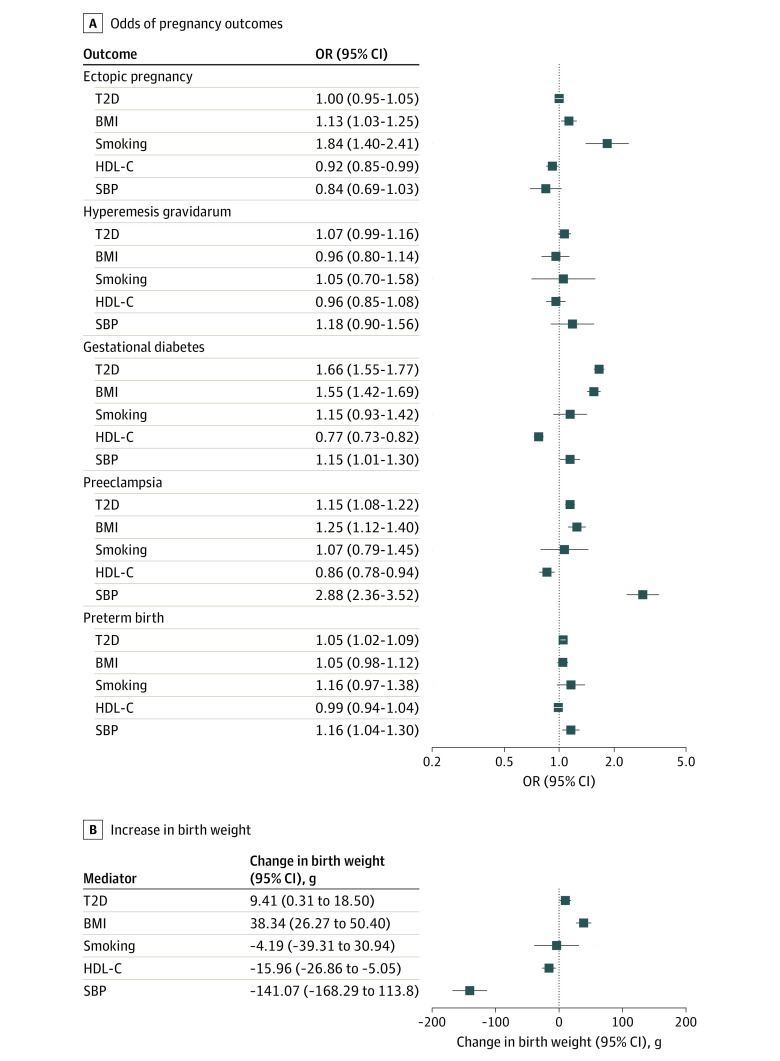
Associations Between Genetically Estimated Cardiometabolic Mediators and Pregnancy Outcomes Results of inverse variance–weighted 2-sample mendelian randomization analyses are shown. Estimates are odds ratios (ORs) for pregnancy outcomes and grams of birth weight per 1-unit increase of the genetically estimated log(odds) of type 2 diabetes (T2D) and 1-SD increase of the other genetically estimated cardiometabolic traits. BMI indicates body mass index; HDL-C, high-density lipoprotein cholesterol level; and SBP, systolic blood pressure.

All genetically estimated cardiometabolic mediators were associated with at least 1 pregnancy outcome. The associations varied greatly between different outcomes (Figure 3). Genetically estimated higher T2D liability was associated with increased risk of gestational diabetes (OR, 1.66 [95% CI, 1.55-1.77]), preeclampsia (OR, 1.15 [95% CI, 1.06-1.22]), preterm birth (OR, 10.5 [95% CI, 1.02-1.09]), and greater birth weight (9.41 [95% CI, 0.31-18.50] g); genetically estimated higher BMI was associated with increased risk of ectopic pregnancy (OR, 1.13 [95% CI, 1.03-1.25]), gestational diabetes (OR, 1.55 [95% CI, 1.12-1.40]), preeclampsia (OR, 1.25 [95% CI, 1.12-1.40]), and greater birth weight (38.34 [95% CI, 26.27-50.40] g); genetically estimated smoking was associated with an increased risk of ectopic pregnancy (OR, 1.84 [95% CI, 1.40-2.41]); genetically estimated higher HDL-C level was associated with a reduced risk of ectopic pregnancy (OR, 0.92 [95% CI, 0.85-0.99]), gestational diabetes (OR, 0.77 [95% CI, 0.7300.82]), preeclampsia (OR, 0.86 [95% CI, 0.78-0.94]), and lower birth weight (−15.98 [95% CI, −26.86 to −5.50] g); and genetically estimated higher SBP was associated with increased risk of gestational diabetes (OR, 1.15 [95% CI, 1.01-1.30]), preeclampsia (OR, 2.88 [95% CI, 2.36-3.52]), preterm birth (OR, 1.16 [95% CI, 1.04-1.30]), and lower birth weight (OR, −141.07 [95% CI, −168.29 to 113.80] g). The sensitivity analyses accounting for pleiotropy supported these associations (eTables 4 to 8 in Supplement 1).

### Mediating Pathways Between Genetically Estimated Level of Education and Pregnancy Outcomes

**Figure 4.**
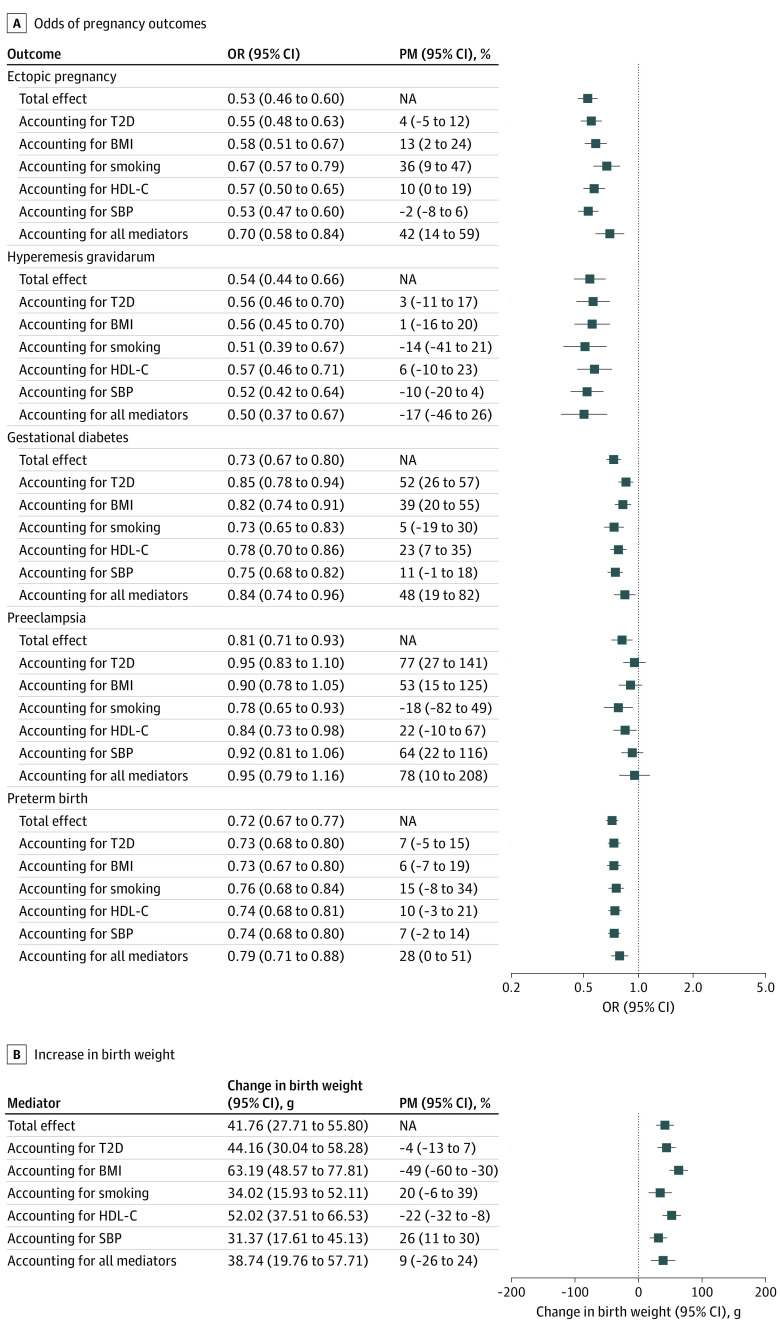
Associations Between Genetically Estimated Educational Attainment and Pregnancy Outcomes After Accounting for Cardiometabolic Mediators Results of mendelian randomization mediation analyses are shown. Estimates are odds ratios (ORs) for pregnancy outcomes and grams of birth weight per a 1-SD increase of genetically estimated years of education (3.4 years). BMI indicates body mass index; HDL-C, high-density lipoprotein cholesterol level; NA, not applicable; PM, proportion mediated; SBP, systolic blood pressure; and T2D, type 2 diabetes.

Figure 4 displays the univariable and multivariable MR estimates of educational attainment on each pregnancy outcome, representing the total and direct associations after accounting for each cardiometabolic mediator alone and all combined, as well as the attenuation in the multivariable MR estimate compared with the univariable MR estimate labeled as the proportion mediated. The attenuation in estimates on adjustment for cardiometabolic traits ranged from almost zero for the association between genetically estimated educational attainment and hyperemesis gravidarum (−17% [95% CI, −46% to 26%]) to most for the association with the risk of preeclampsia (78% [95% CI, 10%-208%]). The degree to which individual cardiometabolic factors attenuated the association between genetically estimated educational attainment and the pregnancy outcomes was largely determined by the association between genetically estimated educational attainment and the mediator and between the genetically estimated mediator and the outcome. For birth weight, the individual cardiometabolic mediators affected associations in competing directions. Thus, there was no net attenuation of the association between genetically estimated educational attainment and birth weight after accounting for all cardiometabolic mediators.

## Discussion

In this 2-sample MR cohort study with mediation analyses, a high level of genetically estimated educational attainment was associated with a reduced risk of every pregnancy outcome assessed and with a higher offspring birth weight. For instance, every genetically estimated 3.4-year increase in education was associated with halved risks of ectopic pregnancy and hyperemesis gravidarum. While the cardiometabolic mediators accounted for one-half of the estimates for gestational diabetes and three-quarters of the estimates for preeclampsia, they did not account for any of the estimate for hyperemesis gravidarum.

### Cardiometabolic Traits and Risk of Adverse Pregnancy Outcomes

In terms of the associations between cardiometabolic traits and pregnancy outcomes, our study generally supports the findings from previous MR studies but also evaluates several novel associations. In a previous MR study, Rogne et al^27^ observed that genetically estimated smoking behavior was associated with the risk of ectopic pregnancy, which was reproduced in the present study. For the other adverse pregnancy outcomes, however, there was no clear association with genetically estimated smoking behavior. While the onset of ectopic pregnancy is a few days after fertilization, the other pregnancy outcomes occur much later in pregnancy. We hypothesize that the null association of smoking with the later-onset pregnancy outcomes may in part be because the genetic associations for smoking behavior were derived from a nonpregnant population.^28^ For genetically estimated HDL-C level, this is the first MR study, to our knowledge, to indicate a potential protective effect on the risk of ectopic pregnancy, and our study supports previous MR studies^29,30^ that have found evidence of a protective association with the risk of gestational diabetes and preeclampsia. While a previous MR study observed no association between genetically estimated HDL-C level and offspring birth weight,^31^ we observed a negative association; the discrepancy may be explained by our study using updated genetic datasets evaluating many more individuals. We found a positive association between genetically estimated BMI and the risk of gestational diabetes, preeclampsia, and birth weight, as previously observed.^29,30,32,33^ We also found that high genetically estimated BMI was associated with an increased risk of ectopic pregnancy, an association that was not present in a previous MR study by Rogne et al^27^ due to smaller sample size. Our study supports previous MR studies reporting a positive association between genetically estimated SBP and risk of preeclampsia—expected due to shared etiology^21^—and lower birth weight^32^ and provides new data on a positive association with risk of gestational diabetes and risk of preterm birth. Last, we found that genetically estimated T2D liability was associated with an increased risk of gestational diabetes, preeclampsia, and higher offspring birth weight, as reported in previous MR studies,^32,34^ and a previously unreported association with increased risk of preterm birth.

### Mediating Pathways Between Level of Education and Pregnancy Outcomes

To our knowledge, only 1 previous MR study^33^ has considered the association between educational attainment and a pregnancy outcome. That study found higher genetically estimated educational attainment to be associated with increased birth weight.^33^ While the investigators did not perform formal mediation analyses, they observed results comparable with those of the main analysis after conducting multivariable MR analyses, including BMI and alcohol consumption. This is in contrast to our finding of an association between genetically estimated educational attainment and birth weight after accounting for BMI. Traditional observational studies^4,35^ have reported that smoking mediates more than one-third of the association of educational attainment with offspring birth weight. This is more pronounced than what our findings suggest, which may be because our smoking variants were imperfect instruments for smoking during pregnancy, as discussed above. Our findings also support previous observations that BMI and smoking mediate in opposite directions, thereby masking socially differentiated healthy fetal growth.^35^

A recent systematic review^1^ summarized the literature of conventional observational studies on mediating pathways between socioeconomic status (including educational attainment) and risk of preterm birth. The individual studies included in the review had discrepant findings but generally reported mediating associations of smoking and BMI comparable with what we found (ie, fairly small mediated associations). When including T2D liability, HDL-C level, and SBP—which were not identified as potential mediators in the review—our analyses support that the 5 cardiometabolic traits combined may explain roughly one-quarter of the effect of genetically estimated educational attainment on the risk of preterm birth.

For preeclampsia and hypertensive disorders of pregnancy, previous studies^2,3^ have observed that the protective association of high educational attainment disappears after accounting for prepregnancy BMI or SBP, which is the same as what we find. Furthermore, smoking behavior has been observed to not mediate any of the association between educational attainment and risk of gestational hypertension,^2^ which is also supported by our findings. What has not previously been reported is our result suggesting that T2D liability may mediate most of the association of genetically estimated educational attainment on risk of preeclampsia.

While educational attainment has previously been observed to be linked to risk of gestational diabetes, little has been reported on potential mediating pathways.^5^ Not surprisingly, given their shared etiology,^36^ our findings support that most of the association of genetically estimated educational attainment may be mediated through T2D liability.

High genetically estimated level of education was associated with a reduced risk of hyperemesis gravidarum, similar to findings in a Norwegian register-based study.^8^ Interestingly, although this was one of the most pronounced associations observed in our study, the analyses suggest that it was not mediated by any of the 5 cardiometabolic traits. In other words, although educational attainment may have a strong effect on risk of hyperemesis gravidarum, this is likely due to factors other than the prenatal cardiometabolic profile.

Finally, while traditional observational studies have observed that high educational attainment is associated with a reduced risk of ectopic pregnancy,^9^ we are the first to show that almost half of this estimate may be explained by cardiometabolic risk factors, smoking in particular. The clinical and public health implications of our findings are described in the eAppendix in Supplement 1.

### Strengths and Limitations

A major strength of our study is that we applied instrumental variable analyses using genetic instruments that allowed for assessment of the causal role of cardiometabolic mediators in the association between genetically estimated educational attainment and risk of several pregnancy outcomes. Due to the random allocation of alleles and because these alleles are static throughout an individual’s life, this design is much less likely to be affected by nondifferential measurement error of the mediator and confounding compared with traditional observational studies.^10,11^ This was further supported by our sensitivity analyses that did not indicate any bias due to pleiotropy. Thus, the association between genetically estimated educational attainment and adverse pregnancy outcomes may be interpreted as an approximation of the unconfounded association between observed (ie, nongenetic) educational attainment and the same outcomes.

This study also has some limitations. A potential limitation is that the genetic associations of the mediators were collected from studies that evaluated nonpregnant populations. This may particularly affect behavioral risk factors such as smoking during pregnancy. A study using data from 2 pregnancy cohorts found that a polygenic risk score for smoking explained 1% to 3% of variance of smoking during pregnancy.^28^ While this is lower than the 4% explained variance in smoking among nonpregnant individuals observed in the GWAS from which the polygenic risk score was based,^37^ it is still clear that genetic instruments of smoking behavior from a nonpregnant population reflect some of the smoking behavior during pregnancy. To our knowledge, our study is the first MR study to evaluate mediating pathways between educational attainment and pregnancy outcomes and the first to evaluate most of the associations among educational attainment, the 5 cardiometabolic traits, and the 6 pregnancy outcomes. To avoid confounding due to population stratification, we evaluated the same genetic ancestry group across all traits.^10^ Consequentially, we strongly encourage studies evaluating other ancestry groups.

## Conclusion

By using causal genetic epidemiological models, our MR cohort study results suggest that interventions aimed at reducing BMI and SBP, reducing T2D and smoking prevalence, and increasing HDL-C level prior to and during pregnancy would lead to reductions in adverse pregnancy outcomes attributable to lower educational attainment. Except for preeclampsia, most of the association of genetically estimated educational attainment with the pregnancy outcomes considered was mediated through other pathways than these cardiometabolic risk factors, which warrants future studies on additional targetable mediators.

## Notes

Supplement 1. **eMethods.** Study Design and Choice of Mediators **eAppendix.** Clinical and Public Health Implications **eTable 1.** Overview of Analyses **eTable 2**. Univariable Mendelian Randomization Analyses of Educational Attainment on Pregnancy Outcomes **eTable 3.** Univariable Mendelian Randomization Analyses of Educational Attainment on Cardiometabolic Mediators **eTable 4**. Univariable Mendelian Randomization Analyses of Type 2 Diabetes on Pregnancy Outcomes **eTable 5**. Univariable Mendelian Randomization Analyses of Body Mass Index on Pregnancy Outcomes **eTable 6**. Univariable Mendelian Randomization Analyses of Smoking on Pregnancy Outcomes **eTable 7**. Univariable Mendelian Randomization Analyses of High-Density Lipoprotein Cholesterol Level on Pregnancy Outcomes **eTable 8**. Univariable Mendelian Randomization Analyses of Systolic Blood Pressure on Pregnancy Outcomes **eFigure.** Schematic Presentation of the Mendelian Randomization Design
eReferences

Supplement 2.
Data Sharing Statement
