# Supplementary material for: Mediating Factors in the Association of Maternal Educational Level With Pregnancy Outcomes: A Mendelian Randomization Study
Source: JAMA Netw Open. 2024 Jan 11;7(1):e2351166. doi: 10.1001/jamanetworkopen.2023.51166 (PMC10784860; doi:10.1001/jamanetworkopen.2023.51166)
Supplement: Supplement 1. — eMethods. Study Design and Choice of Mediators eAppendix. Clinical and Public Health Implications eTable 1. Overview of Analyses eTable 2. Univariable Mendelian Randomization Analyses of Educational Attainment on Pregnancy Outcomes eTable 3. Univariable Mendelian Randomization Analyses of Educational Attainment on Cardiometabolic Mediators eTable 4. Univariable Mendelian Randomization Analyses of Type 2 Diabetes on Pregnancy Outcomes eTable 5. Univariable Mendelian Randomization Analyses of Body Mass Index on Pregnancy Outcomes eTable 6. Univariable Mendelian Randomization Analyses of Smoking on Pregnancy Outcomes eTable 7. Univariable Mendelian Randomization Analyses of High-Density Lipoprotein Cholesterol Level on Pregnancy Outcomes eTable 8. Univariable Mendelian Randomization Analyses of Systolic Blood Pressure on Pregnancy Outcomes eFigure. Schematic Presentation of the Mendelian Randomization Design eReferences [file jamanetwopen-e2351166-s001.pdf]

Supplementary Online Content

Rogne T, Gill D, Liew Z, et al. Mediating factors in the association of maternal educational level with pregnancy outcomes. *JAMA Netw Open*. 2024;6(1):e2351166. doi:10.1001/jamanetworkopen.2023.51166

eMethods. Study Design and Choice of Mediators
eAppendix. Clinical and Public Health Implications
eTable 1. Overview of Analyses
eTable 2. Univariable Mendelian Randomization Analyses of Educational Attainment on Pregnancy Outcomes
eTable 3. Univariable Mendelian Randomization Analyses of Educational Attainment on Cardiometabolic Mediators
eTable 4. Univariable Mendelian Randomization Analyses of Type 2 Diabetes on Pregnancy Outcomes
eTable 5. Univariable Mendelian Randomization Analyses of Body Mass Index on Pregnancy Outcomes
eTable 6. Univariable Mendelian Randomization Analyses of Smoking on Pregnancy Outcomes
eTable 7. Univariable Mendelian Randomization Analyses of High-Density Lipoprotein Cholesterol Level on Pregnancy Outcomes
eTable 8. Univariable Mendelian Randomization Analyses of Systolic Blood Pressure on Pregnancy Outcomes
eFigure. Schematic Presentation of the Mendelian Randomization Design
eReferences

This supplementary material has been provided by the authors to give readers additional information about their work.

© 2024 Rogne T et al. *JAMA Network Open*.

eMethods. Study Design and Choice of Mediators

Study Design

In a two-sample Mendelian randomization (MR) study, for a given single-nucleotide polymorphism (SNP) used as a genetic proxy – or instrument – the SNP-outcome association is divided by the SNP-exposure association to provide an estimate of the exposure-outcome association, often referred to as the Wald ratio.[1] Associations across multiple SNPs are pooled using meta-analytical approaches (Supplementary Figure 1). The steps of a two-sample MR are as follows: First, we identify genetic instruments of the exposure of interest (i.e., educational attainment), next we extract data on how these genetic instruments affect levels of the mediators (e.g., BMI) and outcomes (e.g., preeclampsia), and finally we run analyses as described below.

To limit the risk of confounding due to population stratification, only data from subjects of European ancestry were included.[1]

The summary level data used for this study were publicly available. Data from Steinthorsdottir et al[2] was obtained through application to the Wellcome Sanger Institute (https://ega-archive.org). Data were extracted between December 2022 and April 2023, and analyzed between January 2023 and June 2023.

The number of SNPs included as genetic instruments for educational attainment varied by outcome under study, from 1,294 for birth weight to 1,727 for T2DM.

Choice of Mediators

Based on our previous research on lipids and risk of preeclampsia,[3] we decided to evaluate HDL-C instead of non-HDL-C. To limit the number of potential mediators evaluated, and to focus on more specifically modifiable phenotypes that have strong genetic predictors, we did not evaluate sleep health, diet, or physical activity as mediators.

© 2024 Rogne T et al. *JAMA Network Open*.

eAppendix. Clinical and Public Health Implications

Our study has several important contributions relevant to policy. First, our study strongly supports previous observations that low levels of education are associated with an increased risk of adverse pregnancy outcomes. This is important, because the triangulation of evidence between traditional observational studies and genetic epidemiological studies – with their different sources of bias – underscores that educational attainment is key for healthy pregnancies at the population level. Policies to facilitate access to continuing and higher education for all may thus improve pregnancy health. However, while the largest potential for improved equity is at the societal level, it can be challenging to improve educational attainment, and among those with low educational attainment it is crucial to identify targetable factors that may reduce the risk of adverse pregnancy outcomes. Optimizing all the cardiometabolic traits would considerably lower the risk of ectopic pregnancy, gestational diabetes and preeclampsia, but would have little effect on hyperemesis gravidarum. Subjects with little education and with an otherwise underlying susceptibility to a particular outcome (e.g., previous pelvic surgery leading to an increased risk of ectopic pregnancy) would benefit from more targeted lifestyle changes (e.g., quitting smoking). Finally, for all the evaluated pregnancy outcomes, except preeclampsia, the majority of the effect of educational attainment was mediated through other pathways than the cardiometabolic traits considered; identification of these other factors is important. From an equity perspective, knowledge about the mediating pathways may inform clinicians, public health officials and policy makers where we should prioritize to get closer to equal outcomes in pregnancy health regardless of socioeconomic background.

© 2024 Rogne T et al. *JAMA Network Open*.

eTable 1. Overview of Analyses

Analysis	Notes
Inverse-variance weighted	Main analysis. Greatest statistical power. Assumes all genetic instruments to be valid. Used to estimate the total effect of educational attainment on adverse pregnancy outcomes.
Weighted mode	Provides reliable estimate as long as more genetic instruments estimate the true causal effect than estimate any other quantity. Robust to outlying genetic instruments.
Weighted median	Provides reliable estimate as long as most of the genetic instruments do not have pleiotropic effects. Robust to outlying genetic instruments.
MR Egger	Allows all genetic instruments to have pleiotropic effects as long as the pleiotropic effects on risk of adverse pregnancy outcomes are not correlated with the magnitude of the genetic variants' effects on educational attainment. Sensitive to outlying genetic instruments.
Multivariable analysis	Used to estimate the direct effect of educational attainment on adverse pregnancy outcomes, conditional on the other mediators included in the model.

eTable 2. Univariable Mendelian Randomization Analyses of Educational Attainment on Pregnancy Outcomes

	Number of SNPs	Inverse variance weighted		Weighted mode		Weighted median		MR Egger	
		**OR (95% CI)**	**P-value**	**OR (95% CI)**	**P-value**	**OR (95% CI)**	**P-value**	**OR (95% CI)**	**P-value**
Ectopic pregnancy	1640	0.53 (0.46, 0.60)	2.78E-24	0.47 (0.23, 0.94)	3.29E-02	0.51 (0.42, 0.61)	1.98E-12	0.42 (0.28, 0.62)	1.46E-05
Hyperemesis gravidarum	1640	0.54 (0.44, 0.66)	1.69E-09	1.13 (0.31, 4.05)	8.56E-01	0.64 (0.47, 0.86)	2.96E-03	0.65 (0.34, 1.23)	1.88E-01
Gestational diabetes	1639	0.73 (0.67, 0.80)	2.23E-11	0.73 (0.44, 1.22)	2.28E-01	0.74 (0.65, 0.84)	5.44E-06	0.73 (0.54, 0.98)	3.58E-02
Preeclampsia	1707	0.81 (0.71, 0.93)	2.08E-03	0.76 (0.33, 1.72)	5.04E-01	0.83 (0.68, 1.00)	5.37E-02	0.93 (0.61, 1.42)	7.26E-01
Preterm birth	1689	0.72 (0.67, 0.77)	1.24E-18	1.09 (0.65, 1.81)	7.53E-01	0.78 (0.70, 0.87)	1.68E-05	0.63 (0.50, 0.80)	1.36E-04
		**Grams (95% CI)**	**P-value**	**Grams (95% CI)**	**P-value**	**Grams (95% CI)**	**P-value**	**Grams (95% CI)**	**P-value**
Birth weight	1294	41.76 (27.71, 55.80)	5.59E-09	125.39 (60.66, 190.12)	1.53E-04	55.85 (39.56, 72.15)	1.83E-11	18.57 (-25.42, 62.56)	4.08E-01

CI, confidence interval; OR, odds ratio; SNPs, single-nucleotide polymorphisms.

© 2024 Rogne T et al. *JAMA Network Open*.

eTable 3. Univariable Mendelian Randomization Analyses of Educational Attainment on Cardiometabolic Mediators

	Number of SNPs	Inverse variance weighted		Weighted mode		Weighted median		MR Egger	
		**Beta (95% CI)**	**P-value**	**Beta (95% CI)**	**P-value**	**Beta (95% CI)**	**P-value**	**Beta (95% CI)**	**P-value**
T2DM	1727	-0.60 (-0.66, -0.53)	1.02E-78	-0.49 (-0.80, -0.19)	1.66E-03	-0.58 (-0.64, -0.51)	2.00E-72	-0.73 (-0.92, -0.53)	9.78E-13
BMI	1724	-0.34 (-0.36, -0.31)	5.53E-123	-0.31 (-0.43, -0.20)	1.88E-07	-0.29 (-0.32, -0.27)	1.86E-166	-0.39 (-0.48, -0.30)	3.05E-17
Smoking	1725	-0.39 (-0.41, -0.37)	0	-0.35 (-0.45, -0.25)	4.21E-12	-0.36 (-0.38, -0.34)	6.19E-254	-0.36 (-0.42, -0.31)	2.53E-32
HDL-C	1725	0.21 (0.19, 0.23)	3.93E-99	0.22 (0.14, 0.29)	1.78E-08	0.20 (0.19, 0.22)	3.21E-143	0.29 (0.23, 0.35)	1.73E-19
SBP	1724	-3.12 (-3.53, -2.72)	1.70E-52	-3.73 (-5.63, -1.83)	1.21E-04	-2.96 (-3.40, -2.52)	7.90E-40	-1.97 (-3.24, -0.70)	2.37E-03

Betas reflect standard deviation units for all traits except for T2DM where it reflects log(odds ratio). BMI, body mass index; CI, confidence interval; HDL-C, high-density lipoprotein cholesterol; SBP, systolic blood pressure; SNPs, single-nucleotide polymorphisms; T2DM, type 2 diabetes mellitus.

eTable 4. Univariable Mendelian Randomization Analyses of Type 2 Diabetes on Pregnancy Outcomes

	Number of SNPs	Inverse variance weighted		Weighted mode		Weighted median		MR Egger	
		**OR (95% CI)**	**P-value**	**OR (95% CI)**	**P-value**	**OR (95% CI)**	**P-value**	**OR (95% CI)**	**P-value**
Ectopic pregnancy	237	1.00 (0.95, 1.05)	9.74E-01	0.95 (0.83, 1.08)	4.52E-01	0.98 (0.91, 1.06)	6.16E-01	0.97 (0.86, 1.08)	5.40E-01
Hyperemesis gravidarum	237	1.07 (0.99, 1.16)	1.03E-01	1.07 (0.87, 1.31)	5.41E-01	1.03 (0.90, 1.18)	6.46E-01	1.15 (0.95, 1.39)	1.51E-01
Gestational diabetes	237	1.66 (1.55, 1.77)	1.30E-51	1.59 (1.46, 1.74)	8.19E-21	1.60 (1.50, 1.71)	4.70E-45	1.89 (1.63, 2.19)	6.46E-15
Preeclampsia	249	1.15 (1.08, 1.22)	3.37E-06	1.13 (0.99, 1.29)	7.75E-02	1.14 (1.03, 1.25)	1.00E-02	1.17 (1.02, 1.34)	3.02E-02
Preterm birth	244	1.05 (1.02, 1.09)	2.14E-03	0.99 (0.91, 1.08)	8.03E-01	1.03 (0.98, 1.09)	2.61E-01	1.08 (1.00, 1.17)	3.98E-02
		**Grams (95% CI)**	**P-value**	**Grams (95% CI)**	**P-value**	**Grams (95% CI)**	**P-value**	**Grams (95% CI)**	**P-value**
Birth weight	185	9.41 (0.31, 18.50)	4.26E-02	17.04 (1.45, 32.63)	3.34E-02	10.88 (2.92, 18.84)	7.40E-03	13.66 (-6.58, 33.89)	1.88E-01

CI, confidence interval; OR, odds ratio; SNPs, single-nucleotide polymorphisms.

© 2024 Rogne T et al. *JAMA Network Open*.

eTable 5. Univariable Mendelian Randomization Analyses of Body Mass Index on Pregnancy Outcomes

	Number of SNPs	Inverse variance weighted		Weighted mode		Weighted median		MR Egger	
		**OR (95% CI)**	**P-value**	**OR (95% CI)**	**P-value**	**OR (95% CI)**	**P-value**	**OR (95% CI)**	**P-value**
Ectopic pregnancy	1035	1.13 (1.03, 1.25)	1.31E-02	0.83 (0.49, 1.40)	4.86E-01	1.03 (0.89, 1.20)	6.99E-01	1.05 (0.78, 1.40)	7.68E-01
Hyperemesis gravidarum	1035	0.96 (0.80, 1.14)	6.27E-01	0.98 (0.47, 2.01)	9.46E-01	0.95 (0.74, 1.21)	6.63E-01	0.64 (0.38, 1.09)	1.05E-01
Gestational diabetes	1035	1.55 (1.42, 1.69)	1.59E-23	1.75 (1.17, 2.61)	6.21E-03	1.59 (1.43, 1.78)	1.26E-16	1.59 (1.23, 2.05)	4.27E-04
Preeclampsia	1055	1.25 (1.12, 1.40)	6.94E-05	1.17 (0.65, 2.11)	5.93E-01	1.17 (1.00, 1.38)	5.16E-02	1.15 (0.83, 1.59)	4.12E-01
Preterm birth	1046	1.05 (0.98, 1.12)	1.72E-01	0.86 (0.63, 1.17)	3.39E-01	0.99 (0.89, 1.09)	7.70E-01	0.80 (0.66, 0.97)	2.38E-02
		**Grams (95% CI)**	**P-value**	**Grams (95% CI)**	**P-value**	**Grams (95% CI)**	**P-value**	**Grams (95% CI)**	**P-value**
Birth weight	834	38.34 (26.27, 50.40)	4.73E-10	75.21 (37.02, 113.40)	1.22E-04	43.40 (28.99, 57.82)	3.61E-09	26.35 (-7.60, 60.31)	1.29E-01

CI, confidence interval; OR, odds ratio; SNPs, single-nucleotide polymorphisms.

eTable 6. Univariable Mendelian Randomization Analyses of Smoking on Pregnancy Outcomes

	Number of SNPs	Inverse variance weighted		Weighted mode		Weighted median		MR Egger	
		**OR (95% CI)**	**P-value**	**OR (95% CI)**	**P-value**	**OR (95% CI)**	**P-value**	**OR (95% CI)**	**P-value**
Ectopic pregnancy	139	1.84 (1.40, 2.41)	1.26E-05	1.62 (0.66, 4.00)	2.95E-01	1.71 (1.15, 2.54)	7.58E-03	1.84 (0.59, 5.79)	2.97E-01
Hyperemesis gravidarum	139	1.05 (0.70, 1.58)	8.01E-01	0.43 (0.09, 2.06)	2.93E-01	0.96 (0.52, 1.74)	8.84E-01	0.72 (0.13, 3.94)	7.10E-01
Gestational diabetes	139	1.15 (0.93, 1.42)	1.99E-01	1.13 (0.60, 2.11)	7.12E-01	1.13 (0.86, 1.49)	3.64E-01	0.80 (0.33, 1.95)	6.19E-01
Preeclampsia	144	1.07 (0.79, 1.45)	6.58E-01	1.67 (0.57, 4.93)	3.55E-01	1.16 (0.75, 1.79)	5.01E-01	2.06 (0.62, 6.85)	2.42E-01
Preterm birth	142	1.16 (0.97, 1.38)	9.88E-02	1.07 (0.59, 1.94)	8.36E-01	1.13 (0.88, 1.44)	3.32E-01	1.40 (0.68, 2.88)	3.58E-01
		**Grams (95% CI)**	**P-value**	**Grams (95% CI)**	**P-value**	**Grams (95% CI)**	**P-value**	**Grams (95% CI)**	**P-value**
Birth weight	116	-4.19 (-39.31, 30.94)	8.15E-01	-73.07 (-143.15, -2.99)	4.33E-02	-38.72 (-74.21, -3.22)	3.25E-02	-63.65 (-198.01, 70.71)	3.55E-01

CI, confidence interval; OR, odds ratio; SNPs, single-nucleotide polymorphisms.

© 2024 Rogne T et al. *JAMA Network Open*.

eTable 7. Univariable Mendelian Randomization Analyses of High-Density Lipoprotein Cholesterol Level on Pregnancy Outcomes.

	Number of SNPs	Inverse variance weighted		Weighted mode		Weighted median		MR Egger	
		**OR (95% CI)**	**P-value**	**OR (95% CI)**	**P-value**	**OR (95% CI)**	**P-value**	**OR (95% CI)**	**P-value**
Ectopic pregnancy	1031	0.92 (0.85, 0.99)	2.98E-02	1.05 (0.91, 1.21)	5.23E-01	1.04 (0.92, 1.19)	5.19E-01	0.99 (0.88, 1.11)	8.51E-01
Hyperemesis gravidarum	1031	0.96 (0.85, 1.08)	5.10E-01	1.07 (0.86, 1.33)	5.64E-01	1.08 (0.87, 1.36)	4.83E-01	1.13 (0.94, 1.36)	1.97E-01
Gestational diabetes	1031	0.77 (0.73, 0.82)	4.59E-16	0.98 (0.88, 1.09)	6.87E-01	0.91 (0.82, 1.00)	4.57E-02	0.96 (0.88, 1.06)	4.15E-01
Preeclampsia	1077	0.86 (0.78, 0.94)	1.63E-03	0.84 (0.71, 0.98)	3.09E-02	0.81 (0.70, 0.94)	5.90E-03	0.94 (0.81, 1.09)	4.07E-01
Preterm birth	1066	0.99 (0.94, 1.04)	7.14E-01	1.01 (0.92, 1.11)	7.97E-01	1.01 (0.92, 1.11)	8.69E-01	1.00 (0.92, 1.09)	9.69E-01
		**Grams (95% CI)**	**P-value**	**Grams (95% CI)**	**P-value**	**Grams (95% CI)**	**P-value**	**Grams (95% CI)**	**P-value**
Birth weight	847	-15.96 (-26.86, -5.05)	4.14E-03	-6.60 (-20.53, 7.33)	3.53E-01	-10.77 (-23.51, 1.97)	9.76E-02	-3.84 (-20.36, 12.67)	6.48E-01

CI, confidence interval; OR, odds ratio; SNPs, single-nucleotide polymorphisms.

eTable 8. Univariable Mendelian Randomization Analyses of Systolic Blood Pressure on Pregnancy Outcomes

	Number of SNPs	Inverse variance weighted		Weighted mode		Weighted median		MR Egger	
		**OR (95% CI)**	**P-value**	**OR (95% CI)**	**P-value**	**OR (95% CI)**	**P-value**	**OR (95% CI)**	**P-value**
Ectopic pregnancy	253	0.84 (0.69, 1.03)	9.76E-02	0.70 (0.34, 1.46)	3.47E-01	0.91 (0.70, 1.19)	4.87E-01	0.87 (0.45, 1.66)	6.65E-01
Hyperemesis gravidarum	253	1.18 (0.90, 1.56)	2.31E-01	1.10 (0.41, 2.93)	8.55E-01	1.17 (0.79, 1.74)	4.39E-01	1.40 (0.57, 3.44)	4.62E-01
Gestational diabetes	253	1.15 (1.01, 1.30)	3.68E-02	1.20 (0.81, 1.80)	3.66E-01	1.19 (1.00, 1.42)	5.21E-02	0.97 (0.64, 1.47)	8.73E-01
Preeclampsia	265	2.88 (2.36, 3.52)	2.85E-25	1.96 (0.86, 4.48)	1.11E-01	2.66 (2.05, 3.45)	1.51E-13	4.95 (2.56, 9.59)	3.49E-06
Preterm birth	266	1.16 (1.04, 1.30)	8.03E-03	0.88 (0.60, 1.29)	5.12E-01	1.13 (0.97, 1.32)	1.26E-01	0.97 (0.69, 1.39)	8.86E-01
		**Grams (95% CI)**	**P-value**	**Grams (95% CI)**	**P-value**	**Grams (95% CI)**	**P-value**	**Grams (95% CI)**	**P-value**
Birth weight	230	-141.07 (-168.29, -113.84)	3.12E-24	-127.56 (-172.21, -82.91)	6.12E-08	-124.99 (-147.27, -102.71)	3.98E-28	-176.96 (-264.44, -89.48)	9.83E-05

CI, confidence interval; OR, odds ratio; SNPs, single-nucleotide polymorphisms.

© 2024 Rogne T et al. *JAMA Network Open*.

eFigure. Schematic Presentation of the Mendelian Randomization Design

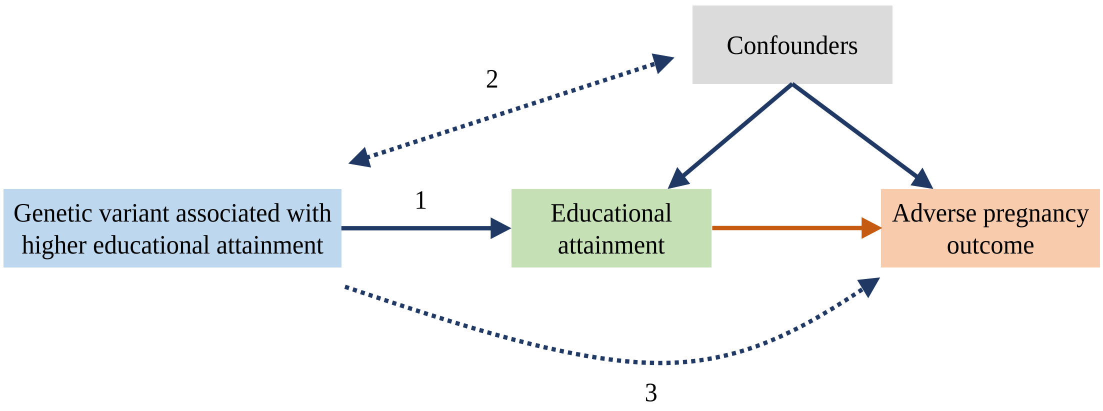

Legend: Red arrow represents the causal effect of interest. For each genetic instrument, the causal effect is estimated by dividing the genetic variant – outcome association by the genetic variant – exposure association. For this estimate to be valid, three main assumptions need to be met: 1) The genetic variant is associated with the exposure; 2) the genetic variant is not associated with outcome via a confounder; and 3) the genetic variant does not directly affect the outcome.

© 2024 Rogne T et al. *JAMA Network Open*.

eReferences

1 Sanderson E, Glymour MM, Holmes M V., Kang H, Morrison J, Munafò MR, *et al.* Mendelian randomization. *Nat Rev Methods Prim* 2022;**2**:6. https://doi.org/10.1038/s43586-021-00092-5.

2 Steinthorsdottir V, McGinnis R, Williams NO, Stefansdottir L, Thorleifsson G, Shooter S, *et al.* Genetic predisposition to hypertension is associated with preeclampsia in European and Central Asian women. *Nat Commun* 2020;**11**:5976. https://doi.org/10.1038/s41467-020-19733-6.

3 Hosier H, Lipkind HS, Rasheed H, DeWan AT, Rogne T. Dyslipidemia and Risk of Preeclampsia: A Multiancestry Mendelian Randomization Study. *Hypertension* 2023;**80**:1067–76. https://doi.org/10.1161/HYPERTENSIONAHA.122.20426.

© 2024 Rogne T et al. *JAMA Network Open*.
