# Supplementary material for: Mediating Factors in the Association of Maternal Educational Level With Pregnancy Outcomes: A Mendelian Randomization Study
Source: JAMA Netw Open. 2024 Jan 11;7(1):e2351166. doi: 10.1001/jamanetworkopen.2023.51166 (PMC10784860; doi:10.1001/jamanetworkopen.2023.51166)
Supplement: Supplement 2. — Data Sharing Statement [file jamanetwopen-e2351166-s002.pdf]

# Data Sharing Statement

Rogne. Mediating Factors in the Association of Maternal Educational Level With Pregnancy Outcomes. *JAMA Netw Open*. Published January 11, 2024.
doi:10.1001/jamanetworkopen.2023.51166

**Data**
**Data available:** No

**Additional Information**
**Explanation for why data not available:** Data already publicly available
